# Mannan-Binding Lectin Attenuates Inflammatory Arthritis Through the Suppression of Osteoclastogenesis

**DOI:** 10.3389/fimmu.2019.01239

**Published:** 2019-06-04

**Authors:** Lijun Dong, Jun Wu, Kai Chen, Jingwen Xie, Youyi Wang, Dantong Li, Yunzhi Liu, Aiping Yin, Yue Zhao, Yunpeng Han, Jia Zhou, Liyun Zhang, Zhengliang Chen, Daming Zuo

**Affiliations:** ^1^Department of Immunology, School of Basic Medical Sciences, Southern Medical University, Guangzhou, China; ^2^Geriatrics Center, General Hospital of Southern Theater Command, PLA, Guangzhou, China; ^3^School of Laboratory Medicine and Biotechnology, Institute of Molecular Immunology, Southern Medical University, Guangzhou, China; ^4^Department of Rheumatology, The Second Affiliated Hospital of Guangzhou University of Traditional Chinese Medicine, Guangzhou, China; ^5^Department of Clinical Laboratory, Guangdong 999 Brain Hospital, Guangzhou, China; ^6^Guangdong Provincial Key Laboratory of Proteomics, Southern Medical University, Guangzhou, China; ^7^Microbiome Medicine Center, Zhujiang Hospital, Southern Medical University, Guangzhou, China

**Keywords:** mannan-binding lectin, arthritis, osteoclastogenesis, receptor activator of nuclear factor-κB ligand, p38

## Abstract

Mannan-binding lectin (MBL) is a vital element in the host innate immune system, which is primarily produced by the liver and secreted into the circulation. Low serum level of MBL is reported to be associated with an increased risk of arthritis. However, the underlying mechanism by which MBL contributes to the pathogenesis of arthritis is poorly understood. In this study, we investigated the precise role of MBL on the course of experimental murine adjuvant-induced arthritis (AIA). MBL-deficient (MBL^−/−^) AIA mice showed significantly increased inflammatory responses compared with wild-type C57BL/6 AIA mice, including exacerbated cartilage damage, enhanced histopathological features and high level of tartrate-resistant acid phosphatase (TRAP)-positive cells. MBL protein markedly inhibited the osteoclast formation from human blood monocytes induced by receptor activator of nuclear factor-κB ligand (RANKL) and macrophage colony-stimulating factor (M-CSF) *in vitro*. Mechanistic studies established that MBL inhibited osteoclast differentiation *via* down-regulation of p38 signaling pathway and subsequent nuclear translocation of c-fos as well as activation of nuclear factor of activated T-cells c1 (NFATc1) pathway. Importantly, we have provided the evidence that concentrations of MBL correlated negatively with the serum levels of amino-terminal propeptide of type I procollagen (PINP) and C-terminal telopeptide of type I collagen (β-CTX), serum markers of bone turnover, in patients with arthritis. Our study revealed an unexpected function of MBL in osteoclastogenesis, thus providing new insight into inflammatory arthritis and other bone-related diseases in patients with MBL deficiency.

## Introduction

Osteoclasts are specialized multinucleated cells derived from precursors in the myeloid/monocyte lineage which resorb bone matrix. Destruction of the joint, in the progression of inflammatory arthritis, is mainly attributed to the osteoclast differentiation and the upregulation of osteoclast-related proteins (1). In osteoclastogenesis, two essential cytokines, receptor activator of nuclear factor-κB ligand (RANKL) and macrophage colony-stimulating factor (M-CSF), are required for the osteoclast formation (2). Sufficient osteoclast differentiation relied on the establishment of specific patterns of gene expression achieved through activation of transcription factors such as PU.1, microphthalmia transcription factor (MITF), c-Fos, c-Myc, NF-kappaB (NF-κB), activator protein-1 (AP-1), and nuclear factor-activated T cells c1 (NFATc1) (3, 4). Among these, NFATc1 is the most highly inducible transcription factor in osteoclast precursor cells and act as a master regulator of osteoclast differentiation through upregulating the osteoclast-specific genes such as cathepsin K (CTSK), tartrate-resistant acid phosphatase (TRAP), matrix metalloproteinase-9 (MMP-9), and osteoclast-associated receptor (OSCAR), thereby promoting the cells fuse to TRAP-positive multinucleated cells (3, 5, 6). Subsequently, osteoclast differentiation results in bone resorption through activation of the RANKL/RANK/osteoprotegerin axis. It is of critical importance to broaden our understanding of the molecular mechanisms that control the osteoclastogenesis during the pathogenesis of inflammatory arthritis.

Mannan-binding lectin (MBL, also called mannose-binding lectin) is a prototypic pattern recognition molecule of the innate immune system, primarily synthesized in the liver and is mostly found in the blood (7, 8). MBL can distinguish between the carbohydrates found on self-glycoproteins and the carbohydrate patterns found on infectious non-self surfaces and initiates the complement cascade through the lectin pathway (7, 9). It is now evident that MBL deficiency is associated with different infectious and autoimmune diseases (10). Previous clinical studies demonstrated that low serum level of MBL predisposes to the development of arthritis and is a risk factor for severity and treatment outcome (11–13). It is noteworthy to mention that we previously observed MBL at high concentrations could suppress the transition of monocytes to dendritic cells (DCs) in the culture condition for DC differentiation (14). Besides, MBL limited the maturation of monocyte-derived DCs induced by lipopolysaccharide (LPS) *via* inhibiting NF-κB activation (15). Indeed, MBL can bind to monocytes and modulates inflammatory cytokine mRNA and protein levels in response to LPS stimulation (16, 17). Therefore, it is possible that MBL might influence the differentiation of monocytes into TRAP-positive osteoclasts and subsequently involved in inflammatory bone destruction, which, however, so far has no evidence to support it.

Adjuvant-induced arthritis (AIA) is a widely used experimental model for the study of inflammatory arthritis (18, 19). A great number of osteoclast precursors, as well as osteoclasts, were generated immediately after the onset of AIA, and these cells were found in the subcompartments of the joints (20). In this study, we used MBL-deficient (MBL^−/−^) mice to generate AIA for evaluating the function MBL on the process of inflammatory arthritis, especially the formation and function of osteoclasts. The results showed that MBL^−/−^ mice were susceptible to AIA and exhibited substantially increased osteoclast formation. *In vitro* study revealed that MBL dose-dependently inhibited the RANKL-induced osteoclast differentiation by suppressing the p38/c-fos/NFATc1 signaling axis. It is noteworthy that we demonstrated that arthritis patients had low serum levels of MBL compared with healthy donors and that concentrations of MBL correlated negatively with the levels of amino-terminal propeptide of type I procollagen (PINP) and C-terminal telopeptide of type I collagen (β-CTX), serum markers for osteoclastic activity, in patients with arthritis. In summary, our findings provide the first line of evidence that MBL might have potential anti-osteoclastogenic effects, which offers insight into the disease mechanisms of inflammatory arthritis and other bone-related diseases, especially in patients with MBL deficiency.

## Materials and Methods

### Patient Samples and Mice

Thirty four serum samples of patients with arthritis were recruited at the Second Affiliated Hospital of Guangzhou University of Traditional Chinese Medicine (Guangzhou, China) from January 2018 to June 2018. Thirty serum samples of healthy donors were also collected as healthy controls (HCs). The patients and controls were well-matched by age and gender. The study was reviewed and approved by the Medical Ethics Committee of Southern Medical University. Before the collection of the blood sample, informed consent for taking part in the study was obtained from each participant.

WT C57BL/6J mice were obtained from the Laboratory Animal Center of Southern Medical University (Guangzhou, China). MBL^−/−^ mice were purchased from the Jackson Laboratory (Bar Harbor, ME, USA). The mice were housed under a specific pathogen-free condition, on a 12-h light-dark cycle, and with food and water *ad libitum*. All animal experiments in this study were approved by the Welfare and Ethical Committee for Experimental Animal Care of Southern Medical University (Approval number: L2016014).

### Reagents

MBL protein was prepared as previously described (16). Recombinant human RANKL (310-01C) and M-CSF (300-25) were purchased from Peprotech (London, UK). Recombinant murine RANKL (315-11) and M-CSF (315-02) were purchased from Peprotech (London, UK). Anti-c-fos antibody (26192-1-AP), anti-GAPDH antibody (10494-1-AP), and anti-CTSK antibody (11239-1-AP) were purchased from proteintech (Chicago, IL, USA). The anti-NFATc1 antibody was purchased from Abclonal Technology (Wuhan, China). Polyclonal antibodies against p38 (8690), phospho-p38 (4511), and Histone H3 (4499) were obtained from Cell Signaling Technology (Cambridge, MA, USA). The TRAP staining kit (387A) was from Sigma-Aldrich (St. Louis, MO, USA). Safranin O-solid green cartilage staining solution (G1317), Hematoxylin-Eosin solution (G4520), Masson's trichrome staining kit (G1340), and Toluidine blue staining solution (G2543) were purchased from Solarbio (Beijing, China).

### Induction of Adjuvant-Induced Arthritis (AIA)

Adjuvant arthritis was induced on day 0 of the experiment by subcutaneous injection of 0.1 ml of complete Freund's adjuvant (CFA) (4 mg/ml heat-killed Mycobacterium tuberculosis, Chondrex, Redmond, WA, USA). Mice were injected with 20 μl of incomplete Freund's adjuvant (IFA) into the knee joint space under general anesthesia on day 14.

### Cell Culture and Osteoclast Differentiation

Human monocytes were purified from EDTA-blood of healthy donors using CD14 Microbeads (Miltenyi Biotec, Bergisch Gladbach, Germany) following the manufacturers' instructions and cultured in α-minimum Eagle's medium (α-MEM) supplemented with 10% heat-inactivated fetal bovine serum (FBS), 1% penicillin/ streptomycin, 50 ng/ml of recombinant human M-CSF and 100 ng/ml of RANKL for indicated days.

### TRAP Staining

To identify osteoclasts, the differentiated cells were fixed in 4% paraformaldehyde for 20 min and then stained with the TRAP staining kit (Sigma-Aldrich) according to the manufacturer's instructions. Dark-red cells containing three or more nuclei were considered TRAP^+^ multinucleated cells. The total number of TRAP-positive osteoclasts was calculated using Osteomeasure software (OsteoMetrics, Inc., Decatur, GA, USA).

### Bone Resorption Assay

The resorptive function of mature osteoclasts was analyzed on bovine bone slices (Immunodiagnostic Systems, London, England). Briefly, cells were cultured in the differentiated medium in the presence or absence of MBL protein for 8 days on bone slices. Then the slices were washed twice with PBS, and the resorption pits were stained with toluidine blue (Sigma-Aldrich) for 5 min. The resorption area was analyzed using the Olympus image system.

### Immunofluorescence Staining

Cells were grown in confocal dishes, fixed in 4% formaldehyde for 15 min at room temperature and permeabilized with 0.25% Triton X-100 for 10 min at room temperature. After blocking with 5% FBS for 1 h, cells were incubated with primary antibodies overnight at 4°C, rinsed, and incubated with fluorescent-conjugated secondary antibodies for 1 h in the dark. Finally, cells were counterstained with 4′,6-diamidino-2-phenylindole (DAPI, Beyotime).

### RNA Isolation and Quantitative Real-Time PCR

Total RNA was extracted using Trizol (TransGene Biotech, Beijing, China) and then transcribed into cDNA using TranScript All-in-One First-Strand cDNA Synthesis SuperMix (TransGene Biotech), as instructed by the manufacturer. Real-time PCR was performed with an Eppendorf Realplex PCR system using TransStart Tip Green qPCR SuperMix (TransGene Biotech). The expression was normalized to the expression of the housekeeping gene GAPDH. The primer sequences used in the experiment are shown in Table 1.

**Table 1:** List of primer sequences used for RT-PCR analysis in this study.

	**Forward primer (5^′^ -3^′^)**	**Reverse primer (5^′^ -3^′^)**
CTSK	ACACCCACTGGGAGCTATG	GACAGGGGTACTTTGAGTCCA
c-fos	CTGTGATCCAAAATCCCTTCAGC	GGTCTGTGGTCTGTACGGAC
DC-STAMP	GGGGCCAGTAGCCAATCTG	CCGTCTCACTATTCACCTGGG
CTR	CCTATCCAACAATAGAGCCCAAG	TGCATTCGGTCATAGCATTTGTA
NFATc1	CACCGCATCACAGGGAAGAC	GCACAGTCAATGACGGCTC
MMP9	TGTACCGCTATGGTTACACTCG	GGCAGGGACAGTTGCTTCT
GAPDH	GGAGCGAGATCCCTCCAAAAT	GGCTGTTGTCATACTTCTCATGG

### ELISA Assay

Mice blood samples were obtained from the carotid artery and centrifuged at 3,500 × g for 15 min, and then the supernatant was collected and set aside at −80°C for serum cytokine analysis. Cytokine levels in the sera were assessed using commercial ELISA kits purchased from eBioscience (San Diego, CA, USA).

### Western Blotting Analysis

Protein lysate was prepared in RIPA buffer (Beyotime, Hangzhou, China) supplemented with protease and phosphatase inhibitor cocktails (Beyotime). Nuclear and cytoplasmic proteins were extracted according to the manufacturer's protocol (Beyotime). Protein samples were fractionated by SDS-PAGE and electrophoretically transferred onto polyvinylidene fluoride (PVDF) membranes (Millipore, Billerica, MA, USA). After blocking with bovine serum albumin (BSA, 5%) for 1 h at room temperature, the membranes were incubated overnight at 4°C with primary antibodies. Subsequently, the membranes were incubated with the horseradish peroxidase-conjugated corresponding secondary antibody for 1 h at room temperature. Finally, detection of the target protein was conducted with enhanced chemiluminescence (Thermo Fisher, Carlsbad, CA, USA) according to the manufacturer's protocol.

### Histological Evaluation

Hind limb samples were collected from arthritis mice and then fixed in 4% paraformaldehyde, embedded in paraffin, and stained with hematoxylin and eosin (H&E), TRAP, Masson's trichrome, safranin O-fast green, and toluidine blue according to the manufacturer's instruction.

For immunohistochemical (IHC) staining, antigen retrieval was performed in a citrate buffer (pH 6.0) at 120°C for 10 min and endogenous peroxidase activity was blocked by exposure to 3% H_2_O_2_ for 15 min. Sections were then incubated with primary antibodies at 4°C overnight. Immuno-reactivity was detected using the corresponding HRP-conjugated secondary antibody and visualized using diaminobenzidine kit (Beyotime Biotechnology, Shanghai, China).

### Micro-Computed Tomography (Micro-CT) Analysis

Knee joints were scanned using a skyscan micro-CT scanner (Bruker, Karlsruhe, Germany) with X-ray beam settings of 50 kV and 50 μA. Trabecular bone regions of interest were selected by highlighting trabecular bone regions for cross-sectional slices of the hind limb and bone architecture determined by quantifying trabecular bone parameters using CTAN software.

### Statistical Analysis

All values were expressed as mean ± SD. One-way ANOVA was used for comparisons among multiple groups. Differences between two groups in the experiments were analyzed by Student's *t*-test. Value of *p* < 0.05 was considered statistically significant.

## Results

### MBL Deficiency Promotes the Progression of Mouse AIA and Associated With Enhanced Inflammatory Mediator

**Figure 1:**
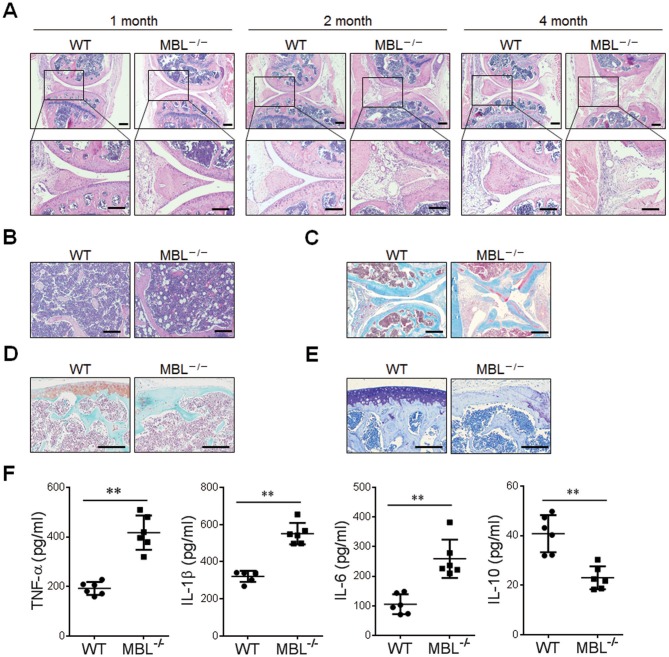
MBL ablation renders mice more susceptible to AIA. WT and MBL^−/−^ mice (*n* = 6) arthritis was induced by immunization with Freund's complete adjuvant and Freund's incomplete adjuvant. **(A)** Histopathological evaluation of the arthritis-induced damage to the knee joints was performed with H&E staining. Bottom panels showed the higher-magnification views of the box area. Scale bars = 200 μm. **(B–E)** Bone destruction and cartilage damage in knee bone from WT and MBL^−/−^ mice were determined after 6 months with adjuvant induction. Osteoporosis was examined by H&E staining in knee bone from WT and MBL^−/−^ mice after 6 months with adjuvant induction **(B)**. Masson's trichrome staining was used to assess collagen deposition **(C)**. Cartilage erosion was assessed by safranin O-fast green staining **(D)**. Toluidine Blue staining was used to qualitatively assess the proteoglycan content in the cartilage **(E)**. Scale bar = 50 μm. **(F)** The serum level of TNF-α, IL-1β, IL-6, and IL-10 from each group of mice 4 months after the primary immunization was measured by ELISA. ^**^p < 0.01. Data are representative of three independent experiments with similar results.

To explore the pathogenic role of MBL in inflammatory arthritis disease, we developed AIA in MBL^−/−^ and paired WT mice. Histological analysis of knee joints after adjuvant injection demonstrated that MBL^−/−^ mice showed severe joint damage compared with WT counterparts (Figure 1A). Besides, MBL^−/−^ mice exhibited more osteoporosis and collagen deposition than WT mice during the development of arthritis (Figures 1B,C). Moreover, there was significantly more destruction of the bone and cartilage in the adjuvant-treated MBL^−/−^ mice than those in WT controls (Figures 1D,E). We also examined and compared the expression of inflammatory cytokines between adjuvant-treated WT and MBL^−/−^ mice. The result showed that aggravated joint damage in MBL^−/−^ mice was accompanied by a significant elevation of pro-inflammatory cytokines (i.e., IL-1β, TNF-α, and IL-6) and reduction of anti-inflammatory cytokine IL-10 in hind paw (Figure 1F).

**Figure 2:**
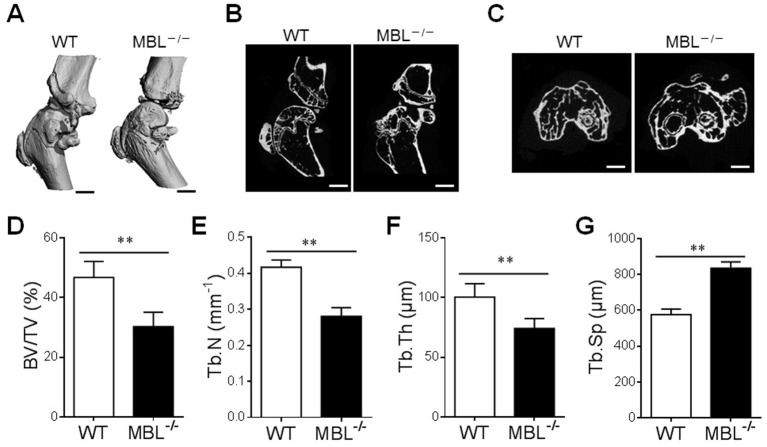
MBL prevents the systemic bone loss of AIA mice. **(A–C)** The hind knee joint was analyzed by micro-CT in AIA mice at months 4. Three-dimensional images of the knee joints of WT and MBL^−/−^ mice (*n* = 6 for each group) were reconstructed **(A)**. Longitudinal mid images. Scale bar = 1 mm. **(B)** and transaxial images. Scale bar = 1 mm. **(C)** from micro-CT analysis of knee joint were presented. Scale bar = 200 μm. **(D–G)** Changes in the morphometric parameters such as bone surface density (BS/TV) **(D)**, trabecular number (Tb.N) **(E)**, trabecular thickness (Tb.Th) **(F)**, and trabecular spacing (Tb.Sp) **(G)** were presented. ^**^p < 0.01. Data shown represent three independent experiments with similar results.

Using the established murine model of AIA, the impact of MBL on bone architecture was evaluated by micro-CT analysis. Qualitative analysis of the three-dimensional reconstruction of the knee joints (Figure 2A) confirmed that MBL had been able to prevent the external focal erosion on the periarticular surfaces. Similarly, longitudinal mid-sections (Figure 2B) and transaxial (Figure 2C) images of knee joint showed a reduction in trabecular bone mass in MBL^−/−^ mice upon adjuvant injection. Compared with WT AIA mice, the MBL^−/−^ AIA mice exhibited decreased bone volume/tissue volume (BV/TV) (Figure 2D), reduced trabecular number (Tb.N) (Figure 2E) and declined trabecular thickness (Tb.Th) (Figure 2F). Concomitantly, the trabecular spacing (Tb.Sp) of MBL^−/−^ AIA mice was significantly higher than that of WT counterparts (Figure 2G). Together, these results indicate a potential protective role of MBL in the pathogenesis of experimental AIA.

### MBL Deficiency Facilitates Osteoclast Formation in Mice AIA

**Figure 3:**
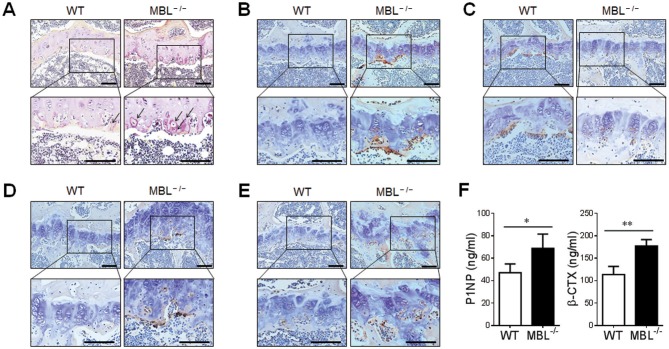
Osteoclast formation is increased in MBL-deficient AIA mice. **(A–E)** The expressions of specific osteoclast markers, TRAP **(A)**, CTSK **(B)**, OPG **(C)** BMP2 **(D)**, and BMP4 **(E)**, in the knee bone tissues from WT and MBL^−/−^ AIA mice were detected 4 months after the primary immunization by immunohistochemical staining. Scale bar = 100 μm. **(F)** Serum activities of P1NP and β-CTX in each group of mice was assessed 4 months after the primary immunization. ^*^p < 0.05, ^**^p < 0.01. Data are representative of three independent experiments with similar results.

Accumulating evidence pointed out that the increased osteoclastic activity is responsible for bone loss or joint destruction during the development of inflammatory arthritis (21). To determine the role of MBL in osteoclastogenesis, TRAP staining was performed on the bone sections isolated from adjuvant-treated WT and MBL^−/−^ mice. The result demonstrated that a massive increase in numbers of osteoclasts in the knee joints from MBL^−/−^ mice compared with those from WT mice (Figure 3A). CTSK is a novel cysteine protease previously reported to be predominantly expressed by osteoclasts (22). Immunolocalization of CTSK revealed a higher amount of CSTK-positive osteoclasts in the joint from adjuvant-treated MBL^−/−^ mice than that from WT controls (Figure 3B). Osteoprotegerin (OPG), produced by osteoblasts, is an essential regulator in osteoclast formation *via* inhibiting both differentiation and function of osteoclasts (23). We observed that OPG expression was significantly lower in MBL^−/−^ AIA mice than WT controls by the immunohistochemical staining (Figure 3C). Besides, bone morphogenetic proteins (BMPs, e.g., BMP2 and BMP4), potent mediators for osteoblast differentiation (24), were strongly over-expressed in the tissues from MBL^−/−^ arthritis mice compared to those from WT counterparts (Figures 3D,E). Moreover, the levels of serum PINP, a biochemical marker of bone formation, was significantly higher in MBL^−/−^ mice with arthritis than WT counterparts (Figure 3F). The adjuvant-treated MBL^−/−^ mice also displayed a markedly increase in the serum level of β-CTX, a marker of bone resorption, compared to WT controls (Figure 3F). Collectively, these data suggest that MBL may modulate the osteoclast formation and activity in the pathogenesis of inflammatory arthritis.

### MBL Suppresses RANKL-Induced Osteoclast Formation *in vitro*

We compared the osteoclast formation in bone marrow cells from WT and MBL^−/−^ mice. Upon cultivation with M-CSF and RANKL, bone marrow cells from MBL^−/−^ mice more efficiently differentiated into mature TRAP-positive multinucleated osteoclasts than those from WT littermates (Supplementary Figure 1A). Consistently, osteoclast cultures of MBL^−/−^ mice displayed an elevated resorption activity (Supplementary Figure 1B). The data indicate that MBL generated by osteoclast precursors and/or mature osteoclasts initiates an autocrine negative feedback loop to regulate osteoclastogenesis.

**Figure 4:**
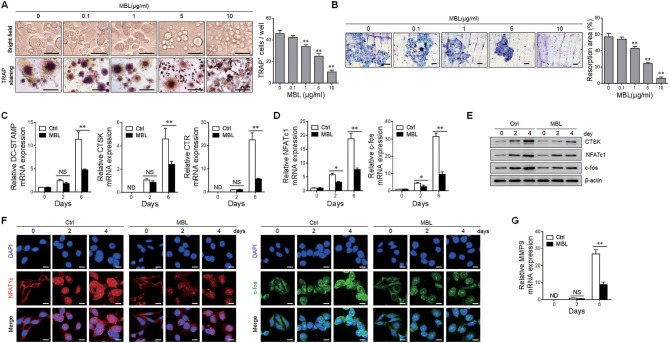
MBL suppresses osteogenesis and osteoclast-specific genes expression *in vitro*. Purified human monocytes were cultured with RANKL (100 ng/ml) and M-CSF (50 ng/ml) in the absence or presence of the indicated concentrations of MBL protein. **(A,B)** Osteoclast differentiation was determined by immunohistochemical staining with TRAP. Scale bar = 50 μm **(A)**. Bone resorption activity was evaluated by measuring the pit area of each well. Scale bar = 100 μm **(B)**. ^**^p < 0.01, compared to the group without MBL protein. **(C)** The mRNA level of osteoclast-specific genes, DC-STAMP, CTSK, and CTR, was detected by quantitative RT-PCR analysis and expressed as a ratio to GAPDH. **(D)** The mRNA expression of novel transcription factors (NFATc1 and c-fos) in osteoclast differentiation proteins was analyzed by real-time RT-PCR. **(E)** The protein level of CTSK, NFATc1, and c-fos was evaluated by immunoblotting analysis. **(F)** The expression and nuclear accumulation of NFATc1 (left panel) and c-fos (right panel) were detected through immunofluorescence analysis. Scale bar = 50 μm. **(G)** Relative mRNA expression levels of MMP9 was measured by quantitative RT-PCR and expressed as a ratio to GAPDH. ^**^p < 0.01, ND, not detectable, NS, not significant. One of the three independent experiments is shown.

Next, we investigated whether exogenous MBL protein affected human osteoclast differentiation *in vitro*. Human purified monocytes were treated with 100 ng/ml of RANKL and 50 ng/ml of M-CSF, which is known to induce osteoclast formation (4, 25), in the presence or absence of varying concentrations of MBL protein. As shown in Figure 4A, MBL treatment significantly reduced the number of TRAP-positive multinucleated osteoclasts. Besides, MBL protein inhibited the bone resorption ability of osteoclasts in a dose-dependent manner (Figure 4B). Consistent with the limitation of osteoclastogenesis, the expression levels of several established osteoclast marker genes, including dendritic cell-specific transmembrane protein (DC-STAMP), CTSK and calcitonin receptor (CTR), were sharply reduced upon MBL administration (Figure 4C). Besides, MBL also showed an inhibitory effect on the induction of c-fos and NFATc1, two of the essential osteoclasts-specific transcription factors, during RANKL-stimulated osteoclast formation (Figure 4D). Western blot analysis further confirmed the reduced protein level of osteoclast marker and transcription factors in MBL-treatment group compared to the control group (Figure 4E). Notably, the nuclear localizations of NFATc1 and c-fos are essential for the osteoclast differentiation (3, 25). We performed immunofluorescence staining assay to confirm whether MBL inhibited the expressions and nuclear localizations of NFATc1 and c-fos in the osteoclasts. The result showed that MBL treatment significantly decreased the expressions and nuclear accumulations of NFATc1 and c-fos in M-CSF and RANKL-stimulated human monocytes (Figure 4F). MMPs are a large group of enzymes responsible for matrix degradation (26). Among the MMPs, MMP-9, uniquely expressed by osteoclasts (27), play a crucial role in joint destruction of inflammatory arthritis (26, 28). Additional study demonstrated that MBL inhibited the expression of MMP-9 in differentiated human osteoclasts (Figure 4G). Taken together, these data indicate that MBL impairs osteoclast differentiation *in vitro*.

### MBL Affects Osteoclastogenesis Through Modulation of p38 Signaling Pathway

**Figure 5:**
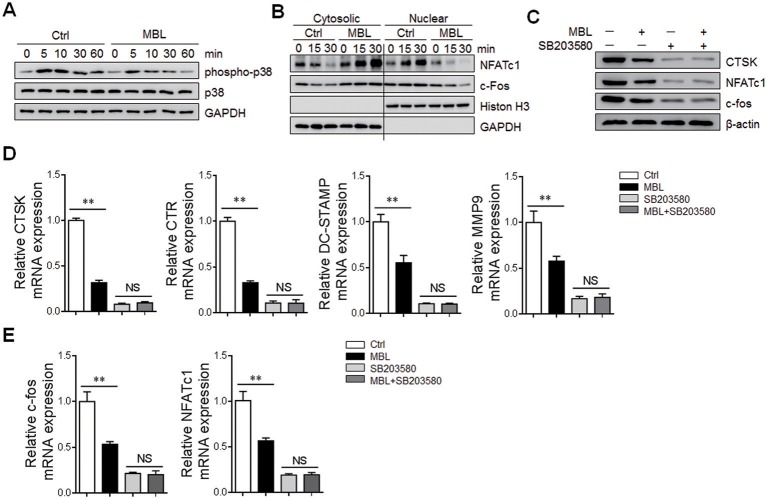
MBL inhibits the osteoclast differentiation *via* downregulation of p38 signaling pathway. The monocytes were treated with RANKL (100 ng/mL) and M-CSF (50 ng/mL) in the presence or absence of 20 μg/ml MBL protein for indicated time points. **(A)** The phosphorylated of p38 were determined by western blot analysis. **(B)** The expression of NFATc1 and c-fos in cytosolic and nuclear fractions of the cells was evaluated by immunoblotting analysis. **(C–E)** Human monocytes were cultured with RANKL (100 ng/mL), M-CSF (50 ng/mL) and MBL protein (20 μg/ml) in the presence or absence of the p38 inhibitor SB203580 (50 nM) for 4 days. Then, the protein levels of CTSK, NFATc1, and c-fos were evaluated by immunoblotting analysis **(C)**. The mRNA levels of osteoclast-specific genes (DC-STAMP, CTSK, and CTR) **(D)** and the novel transcription factors (NFATc1 and c-fos). **(E)** were analyzed by quantitative RT-PCR analysis and expressed as a ratio to GAPDH. ^*^p < 0.05, ^**^p < 0.01, NS, not significant. The data represent three independent experiments with similar results.

All the three MAPK family members (i.e., extracellular signal-regulated kinase (ERK), c-Jun N-terminal kinase (JNK), and p38) are rapidly phosphorylated and activated following RANKL stimulation of osteoclast precursor cells (29). Among them, the role of p38 in osteoclast differentiation and function was extensively investigated (25, 30). Inactivation of the p38 signaling pathway completely blocked the induction of c-fos and NFATc1 with concomitant inhibition of RANKL-induced osteoclastogenesis (25). We, therefore, investigate the activation of the p38 signaling pathway in RANKL-induced osteoclast formation. Enhanced p38 phosphorylation was observed in MBL ^−/−^ mice-derived osteoclasts compared with WT controls (Supplementary Figure 2). Also, MBL protein efficiently inhibited RANKL-induced phosphorylation of p38 in human osteoclast precursors (Figure 5A). Intriguingly, the subsequent nuclear translocations of NFATc1 and c-fos were substantially limited in the presence of MBL (Figure 5B). To further evaluate the effect of p38 activation on MBL-mediated regulation of osteoclast differentiation, osteoclast precursors were pre-treated with p38 inhibitor, SB203580, before RANKL stimulation. The levels of osteoclast markers and osteoclasts-specific transcription factors were strongly reduced and comparable between RANKL-induced osteoclast differentiations with or without MBL incubation upon p38 blockade (Figures 5C–E). These results suggest that the regulation by MBL of osteoclast differentiation is dependent on the p38 signaling pathway.

### The Correlation of Plasma MBL Levels With Disease Severity and Osteoclast Differentiation in Patients With Inflammatory Arthritis

**Figure 6:**
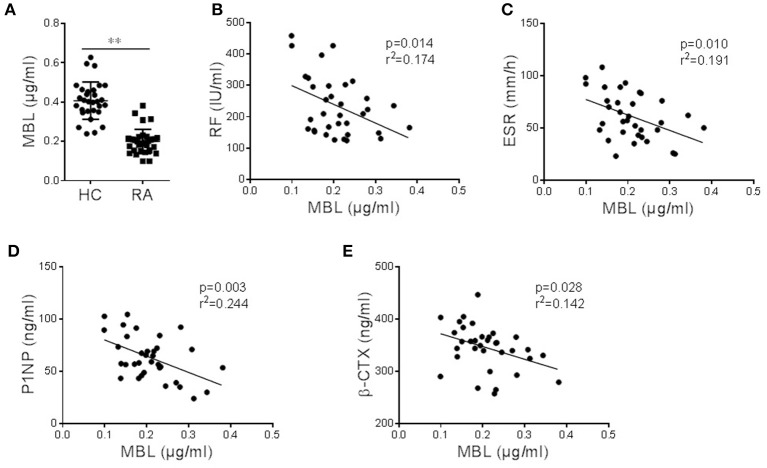
Serum MBL levels are negatively correlated with the severity of arthritis and bone turnover. **(A)** Comparison of serum MBL in healthy controls (HC) (*n* = 30) and arthritis patients (*n* = 34). **(B,C)** Correlation between serum MBL levels with arthritis serological parameters, RF **(B)** and ESR **(C)**, in patients with arthritis. **(D–E)** The relationship of serum MBL concentration with bone turnover biochemical markers, P1NP **(D)** and β-CTX **(E)**, in patients with arthritis.

Rheumatoid arthritis (RA) is characterized by joint inflammation and progressive joint damage, and bone destruction in RA is mainly attributable to the abnormal activation of osteoclasts (1). We, therefore, explored the role of MBL in the progression of RA as well as the process of osteoclastogenesis. In line with previous reports (12, 31), we found that the sera from RA patients showed the MBL serum levels in these patients were significantly lower than those in HCs (Figure 6A). We also analyzed the association of MBL serum level with the disease activity. The results showed that the serum level of MBL was negatively correlated with rheumatoid factor (RF) (Figure 6B) and erythrocyte sedimentation rate (ESR) (Figure 6C). Furthermore, MBL levels were found to be negatively correlated with serum levels of PINP and β-CTX in patients with RA (Figures 6D,E). These data indicate that circulating levels of MBL was significantly associated with the disease activity and osteoclastogenesis in patients with inflammatory arthritis.

## Discussion

MBL is a liver-derived circulating plasma protein, which usually acts as an immunomodulator in the inflammatory response during microbial infection and tissue regeneration (8, 9). It has been reported that MBL deficiency is a significant risk factor for inflammatory arthritis (12, 31). In the present study, we demonstrated that MBL deficiency exacerbated adjuvant-induced inflammatory arthritis in mice, which was associated with a remarkable increase in the formation of osteoclasts. We also assessed the effects of MBL protein on RANKL-induced osteoclastogenesis in human monocytes. Our results indicated that MBL inhibited the RANKL-induced osteoclastogenesis by attenuating the RANKL-mediated p38 pathway activation and inhibiting the level of c-fos and NFATc1 transcription factors. Furthermore, our data demonstrated a significant correlation between the serum level of MBL and bone turnover markers (i.e., PINP and β-CTX) in patients with arthritis.

Cartilage damage is a crucial feature of chronic inflammatory joint diseases (32). Joint damage might result in the release of extracellular matrix (ECM) components (e.g., fibromodulin and aggrecan) into the synovial fluid, which can activate the complement system and form membrane attack complex (MAC) targeting chondrocytes and killing them to cause cartilage damage, thereby continuing self-perpetuating cycle of complement activation and cartilage damage. Wang et al. (33) validated that the expression and activation of complement are abnormally high in human arthritis joints by proteomic and transcriptomic analysis of synovial fluids and synovial membranes from subjects with arthritis. It was thought that complement inhibition would be sufficient to break the cycle of complement activation and cartilage damage and consequent prevent inflammatory arthritis after knee trauma. Indeed, the mice deficient for C3 or factor B were highly resistant to experimental arthritis, indicating that complement activation by both the classical and the alternative pathway acts as a deleterious role in inflammatory (34). Moreover, complement modulation by using C3aR and C5aR antagonists and an anti-C5 blocking antibody was found to be effective in animal studies by ameliorating arthritis or even preventing the disease (35–37). Here, we observed the adjuvant-treated MBL^−/−^ mice exhibited severe joint damage accompanied by the destruction of joint cartilage and bone compared with WT counterparts, indicating that MBL limits the pathogenesis and progression of inflammatory arthritis. It is widely known that MBL-initiated lectin pathway is one of the routes leading to activate the complement cascade. Meanwhile, MBL also performs a regulatory role in the immune system (10). Therefore, we postulated that MBL might be critical in the regulation of inflammatory responses rather than the activation of the complement system in the pathogenesis of inflammatory arthritis.

The effect of MBL on the protection of bone disruption could also be manifested through regulating osteoclast formation. As suggested by our data, MBL has potent activity in globally reducing the expression of specific osteoclast markers in RANKL and M-CSF treated cells, including TRAP, CTSK, CTR, as well as the transcription factor NFATc1 and c-fos. It is noteworthy to mention that osteoclast differentiation contributes to the progressive joint destruction in patients with inflammatory arthritis (4, 38). The involvement of MBL in the processes of coagulation cascade initiation is extensive studied (10). Notably, thrombin inhibits the early stages of RANKL-induced osteoclast differentiation through a direct effect on osteoclast precursors (39). Low MBL level caused by a genetic variation has been reported to be disturbed the particular process during bone healing (40). Herein, we provided the evidence for the first time that MBL could inhibit RANKL-mediated osteoclast differentiation, which strongly expanded our understanding of the pathogenesis of inflammatory arthritis in patients with MBL deficiency. Indeed, our and others' previous reports showed that MBL could modulate the differentiation and function of innate immune cells (i.e., DCs, monocytes, T cells, and mesenchymal cells) in different contexts (14–16, 41, 42). It could be concluded that MBL balancing the differentiation and function of innate immune cells plays an essential role in the maintenance of tissue homeostasis.

The earlier studies have shown that activation of the p38-mediated pathway is indispensable during the osteoclast formation and pharmacological inhibition of p38 activation severely blunts osteoclastogenesis (43, 44). Importantly, p38 has been reported to be crucial for the induction of c-fos and NFATc1 during RANKL-induced osteoclast formation (25). In the present study, we investigated the role of the p38 signaling pathway in RANKL-treated precursors in the presence of MBL protein, and have demonstrated that MBL has an inhibitory effect on osteoclast differentiation *via* downregulation of the p38/c-fos/NFATc1 signaling pathways. The result is consistent with several other observations showing that the regulation of monocytes activation and macrophage polarization by MBL under the inflammatory condition was associated with repression of p38 signaling pathway (45, 46).

Several previous studies reported that the MBL serum levels are significantly reduced in RA patients compared to HC controls (11, 12, 47, 48). A high fraction of RA patients lacked detectable MBL in serum in a long term prospective study, indicating that MBL insufficiency might be a contributing pathogenic factor in RA (48). Low serum MBL was associated with raised RF levels, which therefore could predict poor prognosis in patients with early RA (11). A study about southern Chinese patients with RA determined that a low serum MBL level predisposes to the development of RA and is a risk factor for the development of erosive arthritis (12). Another Brazilian study found that circulating serum MBL levels are significantly lower in RA patients compared to their relatives and controls (47). In contrast, a study done by Saevarsdottir et al. (49) in Iceland showed that patients with RA had strongly higher MBL levels than their close relatives and unrelated controls. Conflicting reports on serum MBL level and the risk of RA may, at least partially, be attributed to the differences in patient cohorts. It is noteworthy that MBL is an acute-phase protein which may transiently increase during inflammatory responses. Indeed, a high serum level of MBL was found to confer an increased risk of overall death and cardiovascular death in RA patients indicating a dual function of MBL in this rheumatic disease (50).

In summary, we demonstrate that MBL deficiency exacerbates AIA through promoting osteoclast differentiation. Importantly, our work elucidates an unknown feature of MBL function in osteoclastogenesis, indicating the association of MBL with bone-related diseases. Our study reveals a new mechanism underlying the pathogenesis of inflammatory arthritis in patients with MBL deficiency and implicates that supplement of MBL may represent a new strategy for the treatment of arthritis, especially in patients with MBL deficiency.

## Data Availability

The raw data supporting the conclusions of this manuscript will be made available by the authors, without undue reservation, to any qualified researcher.

## Ethics Statement

The study was reviewed and approved by the Medical Ethics Committee of Southern Medical University. Before the collection of the blood sample, informed consent for taking part in the study was obtained from each participant. All animal experiments in this study were approved by the Welfare and Ethical Committee for Experimental Animal Care of Southern Medical University.

## Author Contributions

DZ, ZC, and LD designed research. LD, JW, KC, AY, JX, YW, DL, YZ, and YH performed experiments. LD, YL, JZ, LZ and DZ analyzed data. DZ, ZC, and LD wrote the manuscript.

### Conflict of Interest Statement

The authors declare that the research was conducted in the absence of any commercial or financial relationships that could be construed as a potential conflict of interest.

## Abbreviations

IA: adjuvant-induced arthritis
AP-1: activator protein-1
BMP: bone morphogenetic proteins
CFA: complete Freund's adjuvant
CTR: calcitonin receptor
CTSK: cathepsin K
β-CTX: C-terminal telopeptide of type I collagen
DC: dendritic cell
DC-STAMP: dendritic cell-specific transmembrane protein
ECM: extracellular matrix
ERK: extracellular signal-regulated kinase
ESR: erythrocyte sedimentation rate
FBS: fetal bovine serum
H&E: hematoxylin and eosin
IFA: incomplete Freund's adjuvant
IHC: immunohistochemical
JNK: c-Jun N-terminal kinase
LPS: lipopolysaccharide
MAC: membrane attack complex
MBL: Mannan-binding lectin
M-CSF: Macrophage colony-stimulating factor
MITF: microphthalmia transcription factor
MMP: matrix metalloproteinase
NFATc1: nuclear factor of activated T-cells c1
NF-κB: NF-kappaB
OPG: Osteoprotegerin
OSCAR: osteoclast-associated receptor
PINP: amino-terminal propeptide of type I procollagen
PVDF: polyvinylidene fluoride
RA: Rheumatoid arthritis
RF: rheumatoid factor
RANKL: receptor activator of nuclear factor-κB ligand
TRAP: tartrate-resistant acid phosphatase
WT: wild-type.
