# Supplementary material for: Mannan-Binding Lectin Attenuates Inflammatory Arthritis Through the Suppression of Osteoclastogenesis
Source: Front Immunol. 2019 Jun 4;10:1239. doi: 10.3389/fimmu.2019.01239 (PMC6557994; doi:10.3389/fimmu.2019.01239)
Supplement: Supplementary file 1 [file Data_Sheet_1.docx]

**Supplementary Material**

**Supplementary Methods**

**Osteoclast differentiation of primary murine bone marrow cells**

Bone marrow cells isolated from mice were cultured in α-MEM medium supplemented with 30 ng/ml M-CSF and 10% heat-inactivated FBS. After 24 hours, the non-adherent osteoclast precursor cells were resuspended in 10 cm dishes with 30 ng/mL of M-CSF and were allowed to adhere for 2 days. Subsequently, the cells were detached from the dishes, followed by seeded in 24-well plates at a density of 2×10^6^ cells/well and cultured for another 3 days in α-MEM medium containing 10% FBS in the presence of 30ng/ml M-CSF and 100 ng/ml RANKL.

**
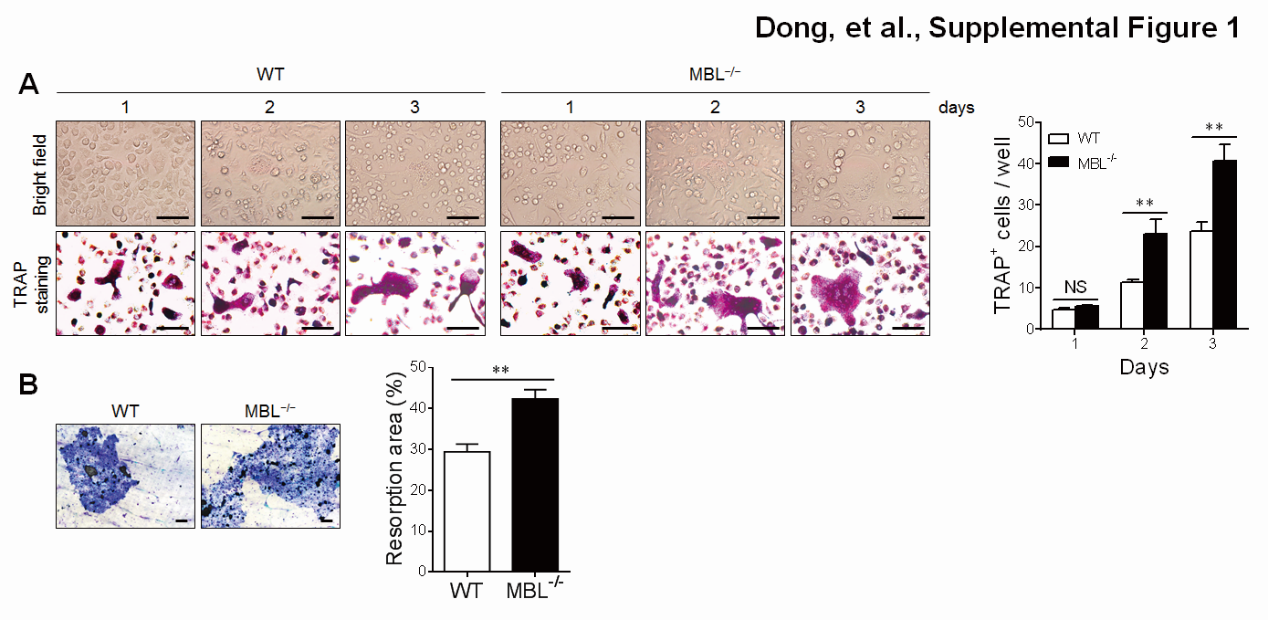
**

**Supplementary Figure 1. MBL directly inhibits murine osteoclastogenesis.**

After incubating with 30ng/ml of M-CSF for 2 days, the bone marrow macrophages were treated with M-CSF (30ng/ml) and RANKL (100ng/ml) for the indicated time periods. (A) Osteoclast differentiation was determined by immunohistochemical staining with TRAP, and the number of TRAP-positive multinuclear osteoclasts was counted. Scale bar=50 μm. (B) Bone resorption activity was evaluated by measuring the pit area of each well on the last day of the differentiation. Scale bar=100 μm. ***p* < 0.01. Data shown represent two independent experiments with similar results.

**
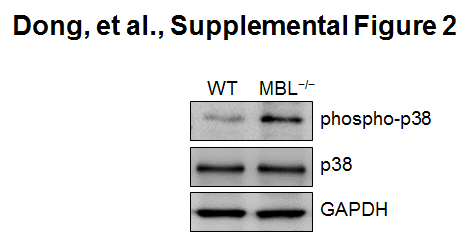
**

**Supplementary Figure 2. MBL deficiency enhances the p38 activation in murine bone marrow-derived osteoclasts**

After incubating with 30ng/ml of M-CSF for 2 days, the bone marrow macrophages were treated with M-CSF (30ng/ml) and RANKL (100ng/ml) for 3 days. The phosphorylation of p38 in the cells was determined by western blot analysis. Data shown represent three independent experiments with similar results.
